# Periodontitis as a risk factor for head and neck cancer

**DOI:** 10.4317/medoral.24270

**Published:** 2020-12-19

**Authors:** Letícia Miquelitto Gasparoni, Fábio Abreu Alves, Marinella Holzhausen, Cláudio Mendes Pannuti, Marianna Sampaio Serpa

**Affiliations:** 1 Department of Stomatology, School of Dentistry, University of São Paulo, Brazil; 2 Department of Stomatology, A.C.Camargo Cancer Center, São Paulo, Brazil

## Abstract

**Background:**

Periodontitis may be associated with the development of head and neck cancer (HNC). A literature review was conducted to understand the possible association between them.

**Material and Methods:**

Articles published in the PubMed database from January 1999 and May 2020 were retrieved. Limitations of the studies and biological mechanisms were discussed.

**Results:**

A total of 4,232 articles were found. Of these, 13 were analyzed according to inclusion criteria. Most papers found some association between periodontitis and HNC, although differences in periodontal evaluation, sample size, study design and tumor sites were observed. Porphyromonas gingivalis appears to increase the chance of both diseases, and it may be one of their main potential risk factors. Genetic predisposition is increased by exposure to environmental factors which can directly induce epigenetic changes that contribute to these diseases.

**Conclusions:**

Understanding the mechanisms related to periodontitis and HNC has increased, however, well-designed clinical studies are needed for better conclusions. Furthermore, the advent of multiple "omic" technologies will help comprehend their possible association.

** Key words:**Periodontitis, head and neck cancer, oral cancer, risk factors, biological factors.

## Introduction

Head and neck cancer (HNC) is the seventh most common cancer in the world. There are about 890,000 new cases annually, with 450,000 deaths every year. The most common histological subtype is squamous cell carcinoma (SCC), and most cases occur in the oral cavity, sinonasal cavity, pharynx, and larynx. Risk factors include smoking, alcoholism, and human papillomavirus (HPV), with the latter being especially involved with the cases of oropharynx. Nevertheless, apart from those well-established risk factors, for oral cancer (OC), approximately 15% of them still cannot be explained, which led to the need to explore other potential risk factors (1,2). Poor oral health, in particular, periodontitis has been hypothesized to be related to the development of HNC (3-15).

**Figure 1:**
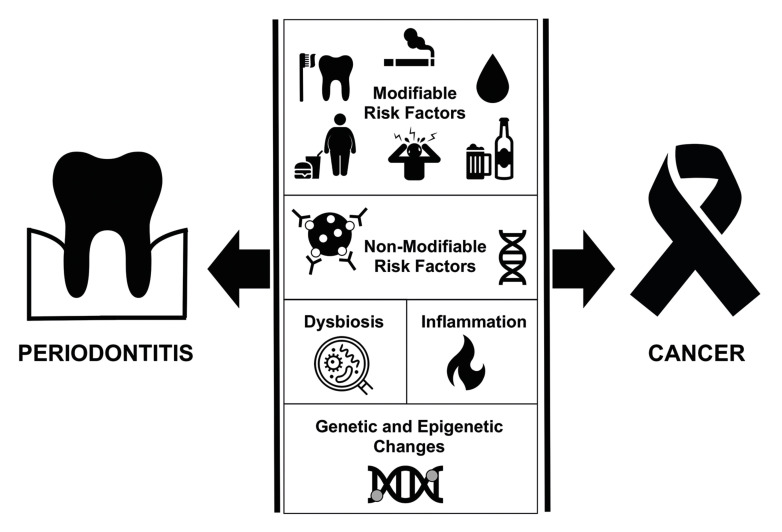
Risk factors for periodontitis and cancer: Modifiable (smoking, diabetes mellitus, psychological factors, and lifestyle factors - such as diet and alcoholism) and non-modifiable risk factors (host response and genetic factors).

Periodontitis is a multifactorial inflammatory disease related with dysbiotic biofilm and characterized by progressive destruction of the tooth-supporting apparatus (16). It is estimated that severe periodontitis affects 538 million people worldwide, and it can lead to tooth loss which negatively impacts quality of life, making it a major public health problem (17). According to Van Dyke and Sheilesh (18), smoking and types 1 and 2 diabetes mellitus are well-established risk factors for periodontitis. In the last decades, several studies have identified alcohol and smoking as the major risk factors of HNC, both associated with poor oral hygiene and, as such, are a possible link between periodontitis and cancer (19). Thus, it is important to understand the risk factors related to periodontitis and HNC and the possible association between the two diseases, in order to propose interventions for prevention therapy, risk reduction and changes in lifestyle (Fig. 1).

In the present study, we reviewed more than 20 years of published data concerning the possible relation between periodontitis and HNC. Highlights from these studies are discussed, including their limitations. The underlying biological mechanisms of periodontitis that could lead to HNC are also discussed aiming to better explore current findings.

## Material and Methods

A search was conducted using the PubMed database addressing studies that analyzed the possible association between periodontitis and HNC or OC over 20 years (between January 1, 1999 and May 20, 2020) in order to understand the overall consensus in the literature concerning this subject. Keywords included: “tooth loss” or “gingival inflammation” or “alveolar bone loss” or “gingivitis” or “periodontitis” or “periodontal” and “tumor” or “carcinoma” or “oral cancer” or “cancer”. Inclusion criteria were: (a) cohort, cross-sectional or case-control studies, (b) full-text articles, (c) and published in English. Case reports were excluded.

## Results

In the total, 4,232 articles appeared in the search. Of these, 20 articles were selected based on the title and abstract. After reading the complete publications, 7 were excluded and 13 remained. For better analysis, we separated HNC from those that were exclusively of OC (Table 1).

**Table 1:**
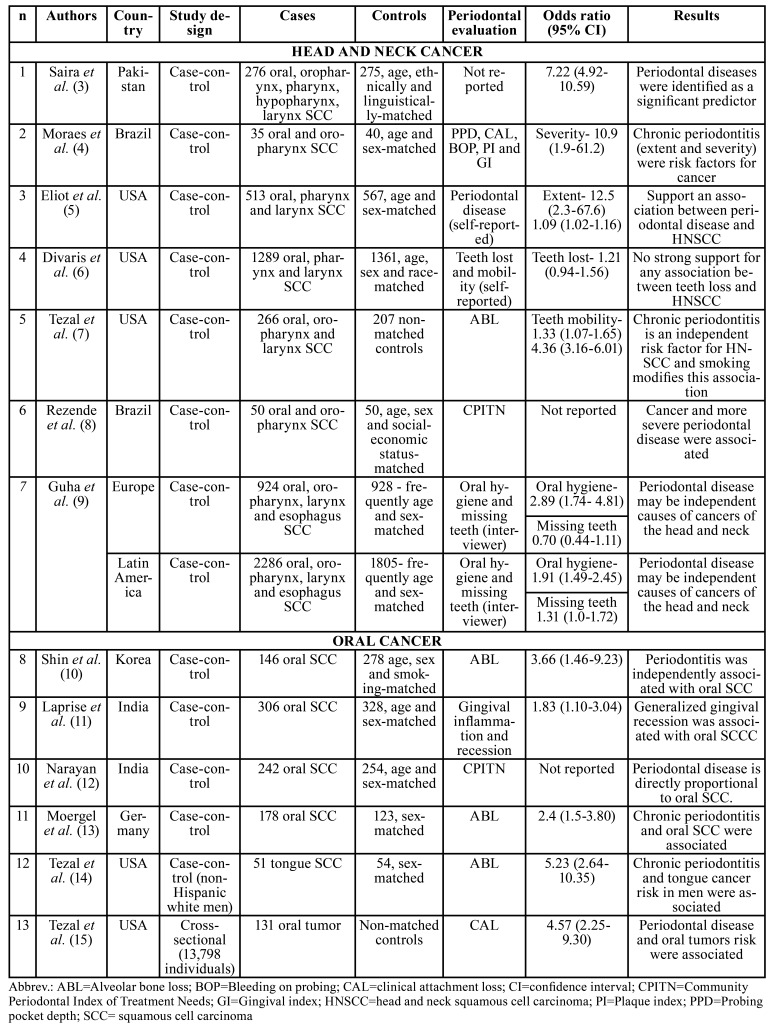
Studies conducted between 1999-2020 that evaluated the association between periodontitis and head and neck or oral cancer.

## Discussion

Overall, most studies reported a positive association between periodontitis and HNC or OC (4,5,7,8,10-15). Tezal *et al*. (14) focused on non-hispanic white men, while other authors (4-13,15) included the entire population, mostly matching the controls with some clinical aspect, such as age, sex, and/or race to diminish bias. Shin *et al*. (10) observed that the relation between periodontitis and OC appears to increase in older (5 times more for patients over 60 years-old) and male patients (6.5 times more than in females), and Tezal *et al*. (14) noticed a 5.23-fold increase in tongue cancer risk with each millimeter of alveolar bone loss (ABL). Furthermore, adjustment of confounding factors (for example, age, sex, education level, smoking, and alcohol drinking) showed that periodontitis is an independent risk factor (10,11).

On the other hand, Divaris *et al*. (6) found no association between tooth loss (oral health assessment) and HNC, showing that there is no consistency between all studies. Furthermore, although Guha *et al*. (9) reported that poor oral health seems to increase HNC risk in Europe, missing teeth in general were not associated with it. Additionally, in Latin America, missing 6 or more teeth seemed to enhance the risk of pharynx and larynx cancer, but no relation was observed with OC, showing variable results. It is important to notice that both Divaris *et al*. (6) and Guha *et al*. (9) used tooth loss as one of the criteria to evaluate periodontitis. This is not the ideal parameter since 30 to 40% of the cases of tooth loss in older subjects are due to cavities and their sequels rather than periodontitis (20).

Smoking and alcohol consumption are well-established risk factors for HNC (2). Thus, to evaluate if periodontitis is indeed a risk factor for HNC these variables were adjusted in most studies (4-7,9-11,14,15). Nevertheless, as both habits (smoking and drinking) are associated with the development of HNC and periodontitis, for future studies that analyze the relationship between periodontitis and HNC it should be interesting to include only patients with HNC who do not smoke or drink as this could lead to more concrete evidence.

A meta-analysis study have reported that periodontitis increases by 2.63-fold the risk for HNC (21). Based on this, it has been speculated that the prevention and treatment of periodontitis could be important to decrease the incidence of HNC and improve its prognosis (7). Nevertheless, to confirm the overall findings well-conducted randomized controlled trials are still needed to be conducted. If this is indeed established, important oral health care policies should be promoted (22).

As for the evaluations used to define the presence of periodontitis, probing pocket depth (PPD) and clinical attachment loss (CAL) are considered the gold standard measurement to identify periodontitis. However, only 1 study used such parameters (4). Probably this occurred because PPD and CAL assessments are more complex methods than the others reported, taking up more time and requiring a proper room to be conducted, which probably made it not feasible. Most authors (n=4) evaluated periodontitis by the presence of ABL using panoramic imaging. However, the quantity of bone loss that defined periodontitis was different between some studies (7,10,13,14). Other periodontal assessments included missing teeth which, as already discussed, may not represent periodontitis, and visual examination or subject questionnaire may be associated with misleading results. Furthermore, studies based on the number of missing teeth may consider that, although unlikely, tooth loss may happen due to cancer and not as the cause of cancer. In addition, especially for the self-reported questionnaires, the educational status of the patient may influence the result as their perception of oral health may be different and result in deceptive conclusions (23). Apart from these different evaluations, one study did not describe the method used to define periodontitis (3) making it difficult to discuss its results as they may have overestimated the relation of periodontitis with OC. For the future, it is important that studies use a validated and optimal measurement of periodontitis, in order to decrease heterogeneity and increase accuracy.

Apart from the several periodontitis assessments that were used which made it harder to properly compare the studies, other limitations included: small sample size for some studies which can overestimate the results; population heterogeneity; differences in study design; varied HNC sites and adjustment of different confounding factors. When taking into consideration all these factors, the results must be interpreted with caution. If periodontitis is indeed a risk for HNC, it is important to understand whether it has a direct or indirect relation with carcinogenesis.

Therefore, it is important to study the biological mechanisms underlying the possible role of periodontitis in HNC for deeper comprehension of the clinical findings. To better discuss these possible mechanisms, this part was divided into 3 topics: (i) microbiological mechanisms, (ii) common genetic factors (polymorphisms) and (iii) immunological mechanisms (epigenetic and inflammatory).

- Microbiological mechanisms

Recently, there was a change in understanding about the origin of microbial diseases. Periodontal diseases can now be explained by polymicrobial synergy and dysbiosis ("a state of imbalance in the relative abundance or influence of species within a microbial community associated with inflammatory disease") (24). Dental biofilm has long been recognized as the initiator of periodontal disease (16). Although the presence of pathogens is a predisposing factor, it is not enough to cause periodontitis. Currently, the most accepted hypothesis is that the oral microbiota dysbiosis interferes with host homeostasis, which leads to periodontitis (24). It is already known that of all microorganisms that colonize the mouth, three specific pathogens are identified as etiological agents in periodontitis: *Porphyromonas *gingivalis**, *Tannerella forsythia* and *Aggregatibacter actinomycetemcomitans* (18). Among them, *P. *gingivalis** is proposed as a keystone pathogen (25), causing in periodontitis dysbiosis and affecting the immune response. This bacterium has also been associated with other diseases, such as diabetes mellitus, cardiovascular diseases, preterm birth, rheumatoid arthritis, and pulmonary disease (26).

A systematic review and meta-analysis evaluated and compared the prevalence of *P. *gingivalis** in cancer patients and showed that although there is no significant correlation between cancer and *P. *gingivalis** (OR, 1.36; 95%CI, 0.47-3.97), this bacterium may be associated with OC. This meta-analysis also highlighted that there were insufficient data associated with sex, smoking, age and alcohol, which are important variables that could potentially influence the malignancy process (27). Geng *et al*. (28) revealed the relationship between *P. *gingivalis**, periodontitis, and cancer using bioinformatic analyses. In this study, it was demonstrated genetic alteration of OC and HNC in response to chronic infection with *P. *gingivalis**.

A review of the possible role of periodontal pathogens in oral carcinogenesis has been published by Perera *et al*. (2) and showed that although recent studies have shown differences in microbial composition between healthy and carcinogenic tissues, they have not been able to agree with specific bacteria or oral microbial dysbiosis patterns implicated in SCC. In this way, there is evidence about the microbiological relationship between periodontitis and HNC, but the mechanisms that are part of this process remain largely unknown.

- Common genetic factors (polymorphisms)

Genes constitute the transcriptionally active part of chromosomes, being structurally divided into introns (non-coding sequences) and exons (coding sequences). DNA sequences may have different variations, and the best known are single nucleotide changes, more commonly called by the abbreviation SNP (single nucleotide polymorphism) that can be found in all regions of a gene (29). A variety of genes may be associated with carcinogenesis. Human microsomal epoxide hydrolase and glutathione s-transferases genes are the most studied polymorphisms regarding the susceptibility to HNC in human. However, there is also an increased risk for HNC for p53 codon 72 Pro/Pro, variants of the ADH gene, and ALDH2 (30).

In 1997, Kornman *et al*. (31) investigated the association between polymorphism of the IL-1 gene and periodontitis severity and found that increased IL-1 production is a strong indicator of susceptibility to periodontitis (OR, 18.9; 95%CI, 1.04-343.05, for ages 40-60 years). After these encouraging results, genetic studies on periodontitis began to focus on polymorphisms. Several polymorphisms were associated with periodontitis, including the Fc-γ receptor, ILs-1,4,6,10,18, TNF-α, vitamin D receptor, cluster of differentiation-14, MMP-1, Toll-like receptor-2 and 4, and COX-2. These studies suggested a connection between periodontitis and genetic variation, however, the relationship between polymorphisms and periodontitis is not always strong due to subtypes of periodontitis and population variations (24).

J. Xu *et al*. (32) evaluated the association between IL-1β polymorphism and cancer. This meta-analysis showed that both the +3954C/T and IL-1β -511C/T polymorphisms might modulate cancer susceptibility. Both periodontitis and HNC are multifactorial diseases, and evidence shows that gene-environment interactions can modulate risk by being associated with multiple risk factors. Genetic predisposition or susceptibility to periodontitis and cancer can be substantially increased by exposure to environmental factors or decreased by control of environmental exposure (33). Thereby, future genetic studies using current strategies will be important to understand periodontitis and HNC.

- Immunological mechanisms (epigenetic and inflammatory)

Classical genetics cannot explain the variety of phenotypes within a population. In this context, epigenetics offers a partial explanation of these phenomena. In 1939, Waddington introduced the term to cite "the causal interactions between genes and their products, which bring the phenotype into existence." Today, epigenetic is related to changes in gene expression that are not coded in the DNA sequence, including chemical changes of DNA and associated proteins, which leads to chromatin remodeling and inactivation or activation of a gene (34). The best-known epigenetic marker is DNA methylation. In 1983, the correlation between cancer and DNA methylation was first demonstrated, where it was observed that the genomes of the cancer cells are hypomethylated in comparison to their normal counterparts (35). In the last decades, it has been observed that carcinogenesis occurs through a multi-stage process in which epigenetic changes in normal cells lead to the generation of highly malignant derivatives. According to Bais (36), smoking, alcohol, HPV, lifestyle changes, and environmental carcinogens could directly induce epigenetic changes and modifications in signaling pathways and enzymes to promote SCC growth and metastasis.

Inflammation is a primordial mechanism in health and disease. The main objective of the inflammatory response is to identify and eliminate factors that affect homeostasis. The inflammatory response consists of four parts: 1) inflammatory inducers; 2) detecting sensors; 3) downstream mediators, and 4) target tissues that are affected (37). This process begins the immune response, involving innate and adaptive immunity. In this part, it seems that an important role is played by the epigenetic regulation of gene expression patterns, which occurs both in the positive regulation of pro-inflammatory cytokines and other signaling molecules to activate a complete immune cell response, as well as in the regulation of anti-inflammatory cytokines. Cytokine genes have been targeted in multiple epigenetic events, such as active histone modifications at regulatory elements and transcriptional activation by loss of DNA methylation (24).

In 1997, Page and Kornman developed the classic pathogenesis model of periodontal disease. While this classic paradigm is still relevant, advances in knowledge have required it to be modified to accommodate new discoveries in the fields of microbiology and immunology. Thus, in the contemporary model of the pathogenesis of periodontitis, there is the transition from health to gingivitis and, finally, to periodontitis (38). The mechanisms involved in this process are related to gene activation pathways and associated with transient changes in the DNA methylation status. Epigenetic changes appear to regulate inflammation-specific genes in periodontitis (24). Recently, some findings suggest that bacteria have the potential to cause alterations in cellular DNA methylation (39). Also, according to Loo *et al*. (40), periodontitis may be associated with DNA hypermethylation, which is related to cancer risk factors. These findings show that cancer and chronic inflammation may have a similar epigenetic pattern, suggesting that DNA methylation may be a link between them.

Epigenetic changes may contribute to cancer, autoimmune and inflammatory diseases, including periodontitis. Recent studies have progressed the understanding of immunological mechanisms, however the knowledge about epigenetic and inflammatory mechanisms to explain the associations between periodontitis and HNC is still limited.

## Conclusions

While it is true that our understanding of the mechanisms related to periodontitis and HNC has increased considerably in recent decades, many challenges remain. More studies are needed to understand these mechanisms and it is expected that with the advent of multiple "omic" technologies (genomics, epigenomics, transcriptomics, proteomics, metabolomics, metagenomics) the understanding of possible association between periodontitis and cancer will be increased. Furthermore, well-designed case-control studies should be encouraged for better conclusions.

Therefore, patients should be encouraged to change their lifestyle and adopt healthy habits (healthy eating, regular physical exercise), eliminate risk factors that may predispose them to cancer (smoking and alcohol), practice good oral hygiene, and visit regularly health professionals. Thus, the health multidisciplinary team must act together to reduce or eliminate potential risks that may affect the oral and systemic overall health of patients.
